# Cross-cultural adaptation process of the "Conversation Analysis Profile for People with Aphasia" to the Portuguese language

**DOI:** 10.1590/S1980-57642014DN83000005

**Published:** 2014

**Authors:** Mariana Ferreira, Diana Oliveira, Ana Correia, Maria dos Anjos Dixe, Sónia Pós de Mina, Anne Whitworth

**Affiliations:** 1,2,3 Speech and Language Therapist, School of Health Sciences, Polytechnic Institute of Leiria, Portugal; 4 Full Professor, School of Health Sciences, Polytechnic Institute of Leiria, Portugal; 5 Associate Professor, School of Health Sciences, Polytechnic Institute of Leiria, Portugal; 6 Associate Professor, School of Psychology and Speech Pathology, Faculty of Health Sciences Curtin University

**Keywords:** aphasia, caregivers, communication, validation study

## Abstract

The Conversation Analysis Profile for People with Aphasia (CAPPA) provides specific information about strengths and weaknesses of the communication between people with aphasia and their family caregiver within a conversational context.

**Objective:**

The aim of this paper was to present the results of the first stages of cross-cultural adaptation of the CAPPA for the European Portuguese language.

**Methods:**

This methodology study describes the translation and back-translation processes, following the recommended steps to that end. In addition, following the consent of one of the original authors, the process of content validation of the CAPPA commenced. The instrument was submitted for assessment before a panel of experts in the area, who constituted the population of this study.

**Results:**

After the translation and back-translation processes, a panel of experts was convened to adapt the Delphi technique. Some questions were excluded on the basis of ambiguity, relevance and potential repetition. Suggestions made by the expert panel were included in a revised version of the tool. 159 items obtained a 100% consensus in relevance, and 157 items were considered suitable by the expert panel, validating the content of the instrument.

**Conclusion:**

The final version will now be applied to the target population in order to carry out the psychometric validation.

## Abstract (pt)

O Perfil de Competências Comunicativas para Indivíduos com Afasia (CAPPA) fornece informações específicas sobre os pontos fortes e fracos da comunicação entre a pessoa com afasia e o seu cuidador familiar dentro de um contexto conversacional.

**Objetivo:**

Apresentar os resultados das primeiras etapas do processo de adaptação transcultural do CAPPA para Português Europeu.

**Métodos:**

Este estudo metodológico retrata o processo de tradução e retroversão do instrumento, seguindo os passos recomendados para esse fim. Além disso, após consentimento de uma das autoras originais, iniciou-se o processo de validação de conteúdo do CAPPA. Para tal, foi necessário submeter o instrumento à avaliação de um painel de peritos na área, que constitui a população deste estudo.

**Resultados:**

Após o processo de tradução e retroversão do instrumento, foi constituído um painel de peritos para desenvolver a Técnica de Delphi. Algumas questões foram excluídas com base na ambiguidade, relevância e redundância. Sugestões feitas pelo painel de peritos foram incluídas na versão final do instrumento. 159 itens obtiveram 100% de consenso na avaliação da relevância e 157 na avaliação da adequação, após análise dos peritos. Sendo assim, o conteúdo do instrumento foi validado.

**Conclusão:**

A versão final vai agora ser aplicada à população-alvo a fim de alcançar a validação psicométrica.

## INTRODUCTION

Aphasia is an impairment of language that occurs as a consequence of an acquired injury in the central nervous system due to stroke, brain skull trauma or tumor.^1^ People with aphasia should not be isolated from their social context but rather, viewed as social beings who continue to communicate with a range of close and distant conversational partners.^2^ This leads to many adaptations in communication, at times manifesting in situations where the caregiver assumes the role of interpreter, reflecting the change in communication skills by the person with aphasia.^3^ At other times, the feelings and attitudes experienced by the person with aphasia can lead to the development of strategies to in crease communication and structure the roles of each member.^4^

Irrespective of the situation, the family of the person with aphasia will experience the need for more information about the nature of the language problem, specifically about the treatment process and ways to maximize efficiency during the communicative interactions.^5^ It is therefore important to work in collaboration with the family and the person with aphasia to identify strategies to facilitate the communication between the family and the person with aphasia.^4^ In working with all involved, the knowledge and strategies learned in therapy will be maximally generalized to other contexts and real-life situations.

A major focus of the research in this area has been on the study of aphasia as a language disability and its subsequent rehabilitation, with a smaller body of literature addressing the needs and difficulties related to disrupted social interactions.^6,7^ Over the past 10-15 years the role of the caregiver has been increasingly recognized and yet research remains relatively limited.^6^

Conversational Analysis (CA) is an approach, developed from ethnomethodology, for analysing the sequential development of interaction between speakers.^7,8^ Through a set of established principles, CA provides a mechanism to analyse whether and how social order is achieved between conversational partners. These principles can also be applied to the identification of problematic issues between two conversational partners in a clinical situation, providing a guide for Therapists to both identify difficulties and structure interventions to facilitate successful communication.^7^

One area in which this is particularly apparent is in the dearth of assessment and evaluation tools to explicitly capture the interaction that takes places between the family and the person with aphasia. One such instrument that has been used widely clinically is the Conversation Analysis Profile for people with Aphasia (CAPPA),^9^ and it is the intention of this study to investigate its potential contribution to the evaluation of interventions involving the person with aphasia and their caregiver.

The main objectives of this tool are to determine the perception of conversational skills of people with aphasia from the viewpoint of the conversational partner and of the person with aphasia; determine the conversational strategies used and their success; and assess changes to conversational styles and communication opportunities since the pre-morbid period. These factors are then interpreted in conjunction with an analysis of conversation between the speakers to gain an insight into the relationship between the perception of conversational partners and what really occurs in a conversational activity.^8^

The Conversation Analysis Profile for people with Aphasia (CAPPA) draws directly on the principles of CA and is designed to provide both an assessment and therapeutic framework. It is comprised of three different sections: a profile of current conversational abilities (Part A), a profile of pre-morbid and current interactional styles and opportunities (Part B) and a conversation analysis between the person with aphasia and their caregiver (Part C). Parts A and B use an interview format to obtain information from the conversational partner and, when possible, from the person with aphasia regarding their perceptions of conversational difficulties. Specifically, Part A contains 26 questions, each including several probes that collect information about how the linguistic deficit is reflected in conversation and the conversational management procedures for (and responses to) repair, topic initiation, turn taking and topic management.

Each question is designed to gather data not only about a particular aspect of the conversation, but also explores:

(a) the response of the conversational partner to a behaviour; and
(b) the conversational success (or otherwise) of this particular response or strategy.

Part B includes 6 questions that aim to compare information on conversational style and patterns pre-morbidly with current interactional styles and opportunities. Part C of the instrument involves the analysis of approximately 10 minutes of a conversation between people with aphasia and their caregiver in a realistic situation.^10^

The CAPPA intends to motivate therapy focused on the promotion of strategies that appears to be effective in the person with aphasia and their caregiver's communication.^11^ The CAPPA does not intend to replace existing linguistic methods of evaluation of communication, but rather emphasizes the interaction between the person with aphasia and their caregiver.^11^

Due to the lack of instruments in the Portuguese language which involve the family caregiver of the person with aphasia, this study intends to present the early stages of cross-cultural adaptation through the translation and back-translation of the CAPPA.

To proceed with the validation of the CAPPA, permission of one of the authors was sought.

This study forms part of a larger project to develop a detailed adaptation of the CAPPA and achieve a semantic, idiomatic and conceptual correspondence between the original tool and the final version in the target language.^12^

The cross-cultural adaptation process should be as viable and valid as the original version.^14^ This paper will report the initial stages of this process.

## METHODS

The cross-cultural adaptation was based on procedures reported by Beaton, Bombardier, Guillemin & Ferraz.^12^

**First translation.** In the first stage, two translations of the original version of CAPPA were carried out into the European Portuguese language. The two translations allowed a comparison between the languages in order to identify any discrepancies in the meaning of words. The translators were bilingual and Portuguese natives but brought different profiles to the process. Translator 1 was aware of the study objectives and was involved in health sciences, while translator 2 was a formal translator who was unaware of the objectives of the study, and was not from a health sciences background; as such, this provided a level of objectivity in providing a different point of view when translating the CAPPA instrument.

During this process, the equivalence of translation of the instrument (item and conceptual equivalence) was respected, since the versions produced retained the same meaning as the original version.

The second stage of the process was based on the original version and the two translations obtained in the first stage. Two specialists in the area of speech-language pathology with a good knowledge of English worked on the three versions. The best translation was selected throughout this stage and, when necessary, by consensus and based on both translations, modifications were made to the construction of phrases to enable a better understanding compared to the original expression (semantic equivalence).

The first version of the CAPPA in European Portuguese language was thus developed.

**Back-translation.** The first version in European Portuguese was forwarded to two bilingual translators who had no prior knowledge of the original instrument.

This process was necessary to determine whether the items of content in the translated version were a faithful reflection of the original version.^13^

The first version was then given to two people with aphasia and their family caregivers, to check the understanding of the terms used as well as the instructions given during the application and the visual appearance (operational equivalence). The criticisms and difficulties reported during the application of thinking aloud were noted. Discussion between the research team and the aphasics took place after each trial . This resulted in changes to the first version of the test, while ensuring that there were no changes in the meaning.

**Cultural adaptation.** Cognisant of the fact that agreement between back-translation and the original instrument does not assure a satisfactory translation, and may only guarantee a consistent translation,^13^ a panel of experts was constituted. This panel included two experienced professionals with 15 years of experience in the area of aphasia and three finals students of Speech-Language Pathology, and the process of thinking aloud was repeated.

This panel compared the two translated versions with the original instrument in English. Further adjustments were then made to achieve a more appropriate version of the instrument in the European Portuguese language. Modifications and adaptations were made, and some expressions of the translated instrument were rewritten. In this sense, with the panel's consensus and ensuring the equivalence of meaning of the instrument, version two of the CAPPA was obtained.

The Panel comprised five Speech and Language Therapists who had practiced for more than eight years in the area of neurological disorders in adults.

## RESULTS AND DISCUSSION

**Review by a Committee of Experts.** In view of the fact that the constructs may differ in the two cultures (English and Portuguese), there was a need to further determine whether the concepts under discussion were interpreted in similar ways.

A panel of experts in the field were therefore convened to develop methods of application of the Delphi technique. The Delphi technique intends to generalize an opinion into a group consensus. The elements which constitute the panel must be experts in the field.^14^ Through several structured questionnaires (rounds), the experts undertake the assessment and review of the instrument.^14,15^ Following this, the process is considered finished when consensus is reached.^16^ A review of literature highlighted a lack of agreement as to the percentage which would determine the validity and the rigour of each item. It was therefore decided to arbitrarily assign 75% of agreement by the panel as the cut-off whereby items were considered valid and consensus reached. If agreement was below 75%, the item was redefined and had to undergo a second round in order to reach the stipulated level of agreement by the panel. Each item was subject to as many rounds as necessary to reach at least the 75% criteria. The experts were chosen according to their involvement and intervention in the area of neurological disorders in adults, with at least eight years working in the area as well as authors of studies or researchers in the same area. All panel members were female. The instrument was submitted to the panel by e-mail and the answers were analyzed anonymously.

The experts were asked to evaluate the instrument according to relevance and suitability. The evaluation was listed as "-1" for "not relevant" and "not suitable"; "0" for "neutral" and/or "with no opinion"; "1" for "relevant" and "suitable".

Overall, the instrument was submitted to the panel of experts three times. As a result of the first round, those items with less than 75% of agreement were rephrased, while other items which had already achieved the 75% were also refined after suggestions for improvement by the experts.

Following the second round, 12 of the 161 items were evaluated as "not relevant" and another five as "not suitable". Eight items were also excluded as they were considered ambiguous, with similar meaning or were already included under other items. This led to a third round where consensus was reached for the remaining items not validated in the previous round.

All the 161 final items were validated, a large number of these reaching 100% of consensus in relevance (159) and suitability (157). The remaining items obtained an opinion consensus of 75%. No items resulted in less than 75% of consensus, therefore it was not necessary to conduct a further round.

## CONCLUSIONS

Based on the process and its outcomes described above, it is possible to affirm that the modified CAPPA instrument is adequate and that the content of the constructs are true to the original version. The major difficulties found during the translation and back-translation processes were in translation of specific linguistic terms and concepts, and not in the content of the constructs. Some terms could not be translated literally, taking into account the cultural context of the Portuguese population and attentive to the fact that the correspondence of a literal term does not necessarily imply the same interpretation in another culture.^16^

Compared to the original version, some items were excluded after being considered ambiguous, irrelevant or representing similar ideas.

The final version obtained is considered suitable for application to a larger sample of the target population and for pursuing psychometric validation of the tool, moving a step closer to determining its contribution to the assessment and intervention of aphasia by Speech and Language Pathologists.

At a later stage, it would be pertinent to create a version of the instrument which can be applied to the Portuguese population not only in Portugal, but also in Brazil.
